# Cadmium Toxicity to *Microcystis aeruginosa* PCC 7806 and Its Microcystin-Lacking Mutant

**DOI:** 10.1371/journal.pone.0116659

**Published:** 2015-01-15

**Authors:** Bin Huang, Shen Xu, Ai-Jun Miao, Lin Xiao, Liu-Yan Yang

**Affiliations:** State Key Laboratory of Pollution Control and Resource Reuse, School of the Environment, Nanjing University, Nanjing, Jiangsu Province, China; University of New South Wales, AUSTRALIA

## Abstract

The adverse effects of microcystin (MC) produced by cyanobacteria have drawn considerable attention from the public. Yet it remains unclear whether MC confers any benefits to the cyanobacteria themselves. One suggested function of MC is complexation, which may influence the bioaccumulation and toxicity of trace metals. To test this hypothesis, we examined Cd toxicity to wild-type *Microcystis aeruginosa* PCC 7806 (WT) and its MC-lacking mutant (MT) under nutrient-enriched (+NP), phosphorus-limited (-P), and nitrogen-limited (-N) conditions. The accumulation of Cd and the biochemical parameters associated with its detoxification [total phosphorus (TP), inorganic polyphosphate (Poly-P), and glutathione (GSH) in the cells as well as intra- and extra-cellular carbohydrates] were quantified. Although the –P cyanobacteria accumulated less Cd than their +NP and –N counterparts, the different nutrient-conditioned cyanobacteria were similarly inhibited by similar free ion concentration of Cd in the medium ([Cd^2+^]_F_). Such good toxicity predictability of [Cd^2+^]_F_ was ascribed to the synchronous decrease in the intracellular concentrations of Cd and TP. Nevertheless, Cd toxicity was still determined by the intracellular Cd to phosphorus ratio (Cd/P), in accordance with what has been reported in the literature. On the other hand, the concentrations of TP, Poly-P, and carbohydrates went up, but GSH concentration dropped down with the enhancement of [Cd^2+^]_F_, indicating their association with Cd detoxification. Although the inactivation of MC peptide synthetase gene had some nutrient and Cd concentration dependent effects on the parameters above, both cyanobacterial strains showed the same Cd accumulation ability and displayed similar Cd sensitivity. These results suggest that MC cannot affect metal toxicity either by regulating metal accumulation or by altering the detoxification ability of the cyanobacteria. Other possible functions of MC need to be further investigated.

## Introduction

*Microcystis* is a freshwater cyanobacterium responsible for harmful algal blooms in rivers, lakes, and reservoirs. One of the major concerns regarding *Microcystis* bloom is its ability to produce the toxin microcystin (MC) [1]. This toxin is a cyclic nonribosomal heptapeptide and has at least 80 congeners. It can strongly inhibit type 1 (PP1) and 2A (PP2A) protein phosphatases and cause serious liver damage. As a secondary metabolite, the functions of MC in cyanobacteria are still obscure. Putative roles include metal complexation, quorum sensing, predator avoidance, and inter-cellular interaction [2, 3]. In our previous study, MC was found not to influence the speciation of four metal ions (Cd, Cr, Cu, and Zn) in the experimental medium [4]. Under this condition, the bioavailability and toxicity of these metals to the freshwater green alga *Chlamydomonas reinhardtii* are still in accordance with the conventional free ion activity model (FIAM) [5]. Namely, the toxicity of these metals is determined by their free ion concentration in the medium. Nevertheless, the case might be different for the MC-producing cyanobacteria, considering the fact that most MC remains inside the cells instead of being released into the environment [6]. Zeng et al. [7] compared the metal sensitivity of both a toxic and a non-toxic environmental isolate of *Microcystis aeruginosa*. The toxic strain is more tolerant to Cd, but both strains display similar sensitivity to Zn. Since these two wild-type (WT) strains might manifest other physiological or biochemical differences, the contribution of MC to their metal sensitivity discrepancy is unclear. A more rigorous manner by which to examine this issue is to compare the metal bioaccumulation or toxicity before and after the knockout of the genes responsible for MC synthesis. Until now, only one such study has been performed by Fujii et al. [8]. They measured the uptake of ferric and ferrous iron by *M. aeruginosa* and its isogenic MC-lacking mutant with no significant differences observed. How metal toxicity (if any) may alter under this condition and whether their findings can be extrapolated to other metals need to be further investigated.

In the present study, we examined Cd toxicity to WT *M. aeruginosa* PCC 7806 and its isogenic MC-lacking mutant (MT) under different nitrogen and phosphorus conditions. As a Lewis acid, Cd is a representative trace metal and belongs to the Class B group [9]. It has different binding affinity to nitrogen-, oxygen-, and sulphur- containing ligands when compared to ferric (Class A) or ferrous iron (Borderline) [8]. Since metal toxicity depends on its concentration and detoxification in the target organisms, we not only measured Cd bioaccumulation but also analyzed the parameters potentially associated with metal detoxification. These parameters include the cellular concentration of total phosphorus (TP), inorganic polyphosphate (Poly-P), low molecular weight (LMW) thiols [glutathione (GSH) and phytochelatins], and MC. The carbohydrates retained inside the organisms as well as those excreted into the medium were also quantified. The toxicity tests were carried out under different nutrient conditions as nutrient concentrations change spatiotemporally in freshwater ecosystems and thus may influence metal accumulation and toxicity in the cyanobacteria [10–13]. The overall objectives of the present study are to elucidate, 1) whether Cd toxicity to WT and MT was different from each other; 2) how Cd accumulation and detoxification ability may change under different nutrient conditions; 3) potential correlations between the first two objectives.

## Materials and Methods

### Cyanobacteria and culture conditions

The freshwater cyanobacteria WT and MT were obtained from the lab of Prof. Dr. Elke Dittmann, Germany. MT is unable to produce any variant of MC because a chloramphenicol resistance gene cassette was inserted into the MC peptide synthetase gene *mcyB* of WT [14]. The two cyanobacterial strains (i.e., WT and MT) were cultured in a modified medium of BG-11 (BG-11_m_) [15]. The chemical components of this medium are listed in Table A of S1 File. The temperature was kept at 25°C with an illumination of 25 μmol photons/m^2^/s in a 12h:12h light-dark cycle. The pH was maintained at 7.1 with the help of 5 mM 3-(N-morpholino)-propanesulfonic acid.

### Toxicity experiments

Six toxicity tests were carried out in total with each strain (WT and MT) tested under three nutrient conditions: nutrient-enriched (+NP), phosphorus-limited (-P), and nitrogen-limited (-N) conditions. BG-11_m_ was the base of the +NP toxicity medium. In contrast, phosphorus or nitrogen was removed from BG-11_m_ to prepare the—P or—N media, respectively. Each toxicity test had eight treatments (A-H) in duplicate (600 mL for each replicate) containing eight total dissolved concentrations (1.00×10^-8^—9.95×10^-6^ M) of Cd ([Cd]_T_, Table B in S1 File). Ethylenediaminetetraacetate (EDTA, final concentration—10 μM) was applied to keep the free Cd ion concentration ([Cd^2+^]_F_, 1.00×10^-13^—1.21×10^-8^ M) constant [7]. Although EDTA complexation reduced the toxicity of total Cd, the [Cd^2+^]_F_-based toxicity results were unaffected according to FIAM. The MINEQL+ software package (Version 4.5 from Environmental Research Software, Hallowell, ME, USA) was employed to calculate [Cd^2+^]_F_ of each treatment (Table B in S1 File). All media were prepared one day before the toxicity assays and left undisturbed overnight at 25 °C to reach chemical equilibration. Then an aliquot of 0.5 mL from each replicate was taken and acidified with ultrapure concentrated HNO_3_ (final concentration, 3.5% w/v). Thereafter, its [Cd]_T_ was measured by a graphite furnace atomic absorption spectrophotometer (GFAAS, Thermo Fisher Scientific Inc., Waltham, MA, USA) [4]. Polypropylene beakers and polycarbonate bottles were used throughout the experiment to minimize Cd loss onto the container wall. All containers were soaked in 1 N HCl for at least 24 h and rinsed with Milli-Q water for 6 times before being employed in the present study.

The cyanobacteria to be used in the +NP toxicity test were cultured in BG-11_m_ until they arrived at the mid-exponential growth phase (~2×10^6^ cells/mL). Then they were harvested by centrifugation (3900×g, 10 min), rinsed with fresh BG-11_m_, and resuspended into the +NP media. The +NP cells were further incubated in the—P or-N media for 4 d or 3 d in the respective experiments before resuspension. Both the—P and—N cells grew much slower and contained less phosphorus or nitrogen than the +NP cells. Each toxicity test lasted 72 h with four time points (0, 24, 48, and 72 h). At each time, the average density and diameter of the cells were measured by a Z2 Coulter Counter (Beckman Coulter, Inc., CA, USA). Then the cell specific growth rate μ was calculated according to the methodology described by Miao and Wang [16]. Since the cell size might change with nutrient and Cd concentrations in the medium [17, 18], all parameters were normalized to cell volume when applicable. After 72-h exposure, 30 mL aliquot was filtered through a 1.2 μm polycarbonate membrane (Isopore, Merck Millipore, Billerica, MA, USA) and rinsed with 0.1 mM EDTA to remove the cell-surface-adsorbed Cd. The filter was then digested in 0.5 mL concentrated ultrapure nitric acid for the determination of intracellular Cd concentration ([Cd]_intra_) by GFAAS. Cadmium concentration in the filtrate was also measured to see whether the sum of extracellular and intracellular Cd was equal to the total amount of Cd initially added into the medium. On the other hand, cellular concentrations of TP and Poly-P were quantified by the molybdenum blue method [19, 20]. For this purpose, a 100 mL aliquot from each replicate was filtered through a combusted GF/F membrane (Whatman, GE Healthcare Bio-Sciences Corp., Piscataway, NJ, USA), which was then ground in 9 mL deionized water by a glass pestle. Part of the slurry (4 mL) was taken for the measurement of TP [20]. The remaining slurry was then extracted by 0.3 N NaOH. Total and soluble reactive phosphorus in the extract were determined colorimetrically [19, 20] and their difference is defined as Poly-P.

Being the most abundant MC variant, cellular concentration of MC-LR (CAS #, 101043–37–2) ([MC-LR]_cell_) was analyzed via the method of Ramanan et al. [21]. Briefly, a 100 mL aliquot was filtered through a combusted GF/F membrane and freeze-dried at the end of each toxicity test. MC-LR in the cells was then extracted by a 75% v/v aqueous solution of methanol and purified through a preconditioned Sep-Pak C18 Vac cartridge (0.5g/3mL, Waters Corp., Milford, MA, USA). Thereafter, MC-LR was determined by HPLC (1200 series, Agilent Technologies, Santa Clara, CA, USA) (ZORBAX Eclipse XDB-C18 column, 4.6 × 250 mm) through a DAD detector. Another 30 mL sample from each replicate was passed through a combusted GF/F membrane. The carbohydrates in the filter and filtrate were reduced to alditol by potassium borohydroxide and oxidized to formaldehyde by periodate. Subsequently, the concentration of cellular carbohydrates ([-CHO]_cell_) and the concentration of mono- ([-CHO]_mono_) and polysaccharide ([-CHO]_poly_) excreted by the cyanobacteria were determined spectrophotometrically with the color reagent of 3-methyl-2-benzothiazolinone hydrozone hydrochloride [22, 23]. Cellular concentration of LMW thiols including GSH and phytochelatins was also quantified fluorometrically through HPLC (Supelco Discovery RP Amide-C16 column, 4.6 × 250 mm; 380 nm excitation and 470 nm emission) after derivation by the fluorescence tag monobromobimane [24]. Since all disulfides in the sample were reduced by excess dithiothreitol before fluorescence derivation, both oxidized and reduced thiols were measured through this method. In order to compare the difference between different nutrient-conditioned WT and MT at various Cd levels more easily, the cellular concentrations of total phosphorus (TP), inorganic polyphosphate (Poly-P), low molecular weight (LMW) thiols, and different types of carbohydrate concentration were normalized to levels detected in the WT strain at the lowest respective Cd concentration (Treatment A). Nevertheless, the absolute values of these parameters are provided in S1 File.

### Statistical analysis

Any significant difference (accepted at *p* < 0.05) was based on the results of one-way or two-way analysis of variance (ANOVA) with post-hoc multiple comparisons (Tukey for equal variance or Tamhane for unequal variance) (SPSS 11.0 by SPSS, Chicago, USA). The only exception to the SPSS statistical analysis was the comparison of various dose-response curves in the six toxicity tests through Graphpad Prism (version 5.01 from GraphPad Software, Inc., La Jolla, CA, USA). The dose-response curves were simulated by the Logistic model, based on which the [Cd^2+^]_F_-based median effect concentrations (EC50s) were obtained. Regarding the large number of samples to be analyzed, two independent replicates were applied in all treatments of the six toxicity tests above. This arrangement is acceptable as small variation was always observed between the two replicates of each treatment (see next section).

## Results and Discussion

### Cd bioaccumulation and toxicity

**Figure 1:**
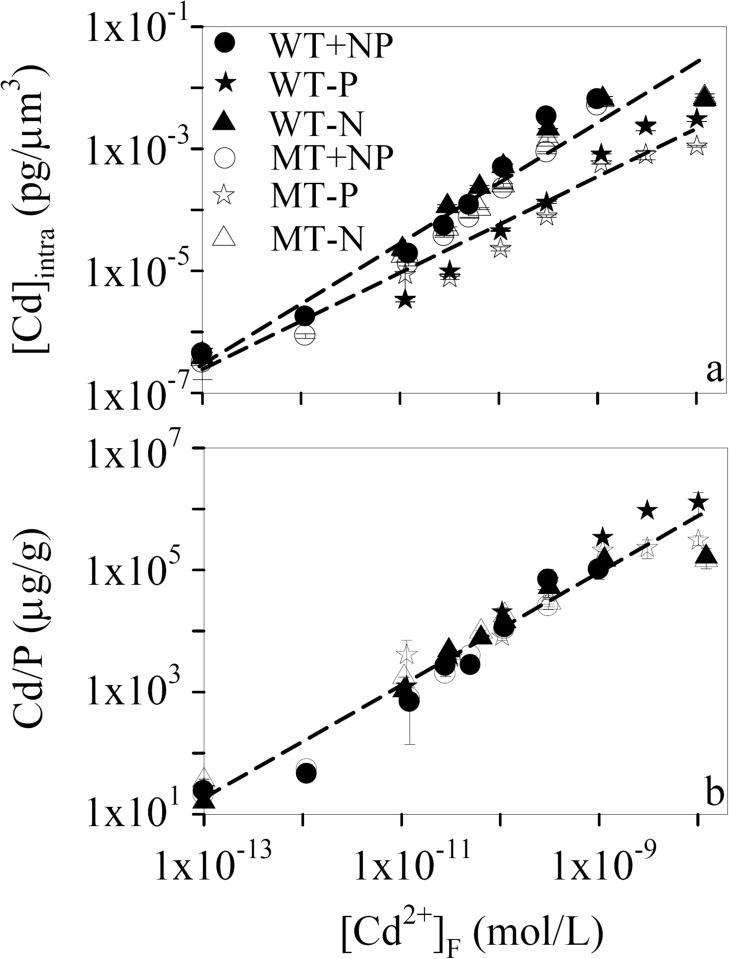
Variation of (a) intracellular Cd concentration ([Cd]_intra_) and (b) intracellular Cd to phosphorus ratio (Cd/P) with free Cd ion concentration of the experimental media ([Cd^2+^]_F_, 1.00×10^-13^—1.21×10^-8^ M) in the nutrient-enriched (+NP, circle), phosphorus-limited (-P, star), and nitrogen-limited (-N, triangle) toxicity tests for *Microcystis aeruginosa* PCC 7806 (WT, filled symbols) and its MC-lacking mutant (MT, open symbols). Dashed lines are the simulation of the positive relationship between [Cd^2+^]_F_ and [Cd]_intra_ or Cd/P by the Freundlich isotherm. Data are mean ± standard error (n = 2)

At the end of each 72-h toxicity test, [Cd]_intra_ went up linearly with [Cd^2+^]_F_ first in the log-scale (Fig. 1a). Then it leveled off when [Cd^2+^]_F_ was higher than 3.06×10^-10^, 1.09×10^-9^, and 3.15×10^-10^ mol/L in the +NP,-P, and—N toxicity tests, respectively. Further, Cd bioaccumulation ability of WT and MT was comparable to each other (*p* > 0.05, two-way ANOVA). Accordingly, [Cd]_intra_ ranged from 3.84×10^-7^ to 6.54×10^-3^ pg/μm^3^ for WT and from 3.61×10^-7^ to 6.94×10^-3^ pg/μm^3^ for MT in the—N toxicity tests. Zhou et al. [25] cultured the same cyanobacterial species in BG-11 with [Cd^2+^]_F_ of 10^-11^—10^-10^ M for 48 h. Assuming the cell volume of this cyanobacterium is 25 μm^3^, its [Cd]_intra_ ranges from 1.72×10^-4^ to 7.84×10^-4^ pg/μm^3^, similar to what we found (1.89×10^-5^—4.87×10^-4^ pg/μm^3^) herein.

More interestingly, the—N cyanobacteria exhibited no significant differences (*p* > 0.05, two-way ANOVA) in [Cd]_intra_, compared to their +NP counterparts (Fig. 1a). Under this condition, all data points in the +NP and—N toxicity tests with both cyanobacterial strains can be fitted to a single Freundlich isotherm ([Cd]_inta_ = 2.5×10^6^×[Cd^2+^]_F_, *r* ^2^ = 0.95, *p* < 0.01). By contrast, [Cd]_intra_ of the—P cells ([Cd]_inta_ = 4.4×10^3^×([Cd^2+^]_F_)^0.8^, *r* ^2^ = 0.94, *p* < 0.01) was approximately one order of magnitude lower than those of the other two nutrient-conditioned cyanobacteria. As metal accumulation is determined by its uptake and efflux, the lowered [Cd]_intra_ of the—P cells might result from the weakened Cd uptake accompanying the reduced phosphorus assimilation under the—P condition [11]. Moreover, the higher accumulation of Cd under the +NP and—N conditions may reflect the potential effects of phosphorus (Poly-P) in metal detoxification, which will be discussed later. In fact, reduced metal uptake under the—P condition is commonplace for freshwater phytoplankton (e.g., *C. reinhardtii*, *M. aeruginosa*, and *Scenedesmus obliquus*) [11–13]. An increase in phosphate concentration of the experimental medium from 0.5 to 50 μM induces the accumulation of Cd and Zn by 18 and 5 times, respectively, for *C. reinhardtii* [13]. Nevertheless, such phenomenon was not observed in marine phytoplankton, which have similar metal accumulation under the +NP and—P conditions [24, 26, 27]. Besides their different Cd accumulation phenotypes in response to phosphorus limitation, the freshwater and marine phytoplankton also perform differently under the—N condition. Namely, [Cd]_intra_ remains unaffected for freshwater phytoplankton, but declines markedly for marine phytoplankton when they are nitrogen-limited [24, 26]. The unchanged [Cd]_intra_ under the—N condition in the present study (Fig. 1a) might be attributed to the negligible effects of nitrogen limitation on Cd uptake as reported by Yu and Wang [12, 13]. Conversely, the lowered Cd accumulation by the-N marine phytoplankton was proposed to be caused by the down-regulated synthesis of metal transporters on the plasma membrane, considering the fact that nitrogen is an important component of these transporters [24, 28]. The underlying mechanisms for such striking difference between freshwater and marine phytoplankton need to be further elucidated, especially from an evolutionary perspective.

**Figure 2:**
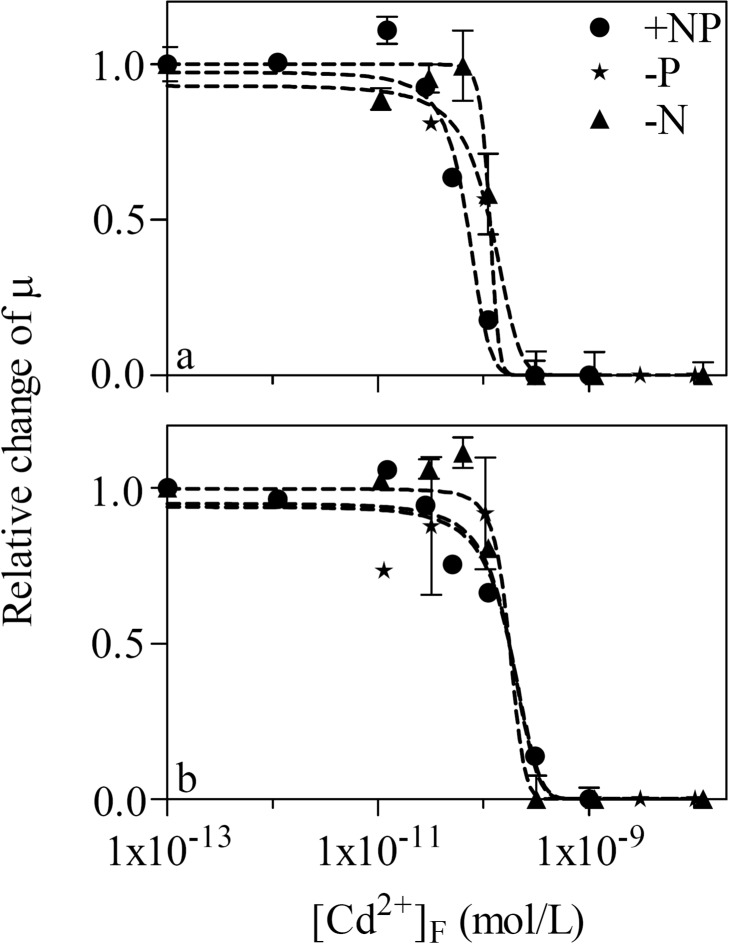
Relative change of the cell specific growth rate (μ) in treatments with different free Cd ion concentration ([Cd^2+^]_F_, 1.00×10^-13^—1.21×10^-8^ M) as compared to its counterpart in the respective control treatment of the nutrient-enriched (+NP, circle), phosphorus-limited (-P, star), and nitrogen-limited (-N, triangle) toxicity tests for (a) *Microcystis aeruginosa* PCC 7806 (WT) and (b) its MC-lacking mutant (MT). Dashed lines are dose-response curves simulated by the Logistic model. Data are mean ± standard error (n = 2)

As a result of Cd accumulation, the cell growth of WT and MT was significantly (*p* < 0.05, one-way ANOVA) suppressed at higher [Cd^2+^]_F_. Accordingly, μ stayed constant (0.35–0.41 d^-1^) in the first four treatments of the +NP toxicity test for WT. Thereafter, it declined to 0.24 d^-1^ with [Cd^2+^]_F_ of 5.04×10^-11^ M and approached zero after [Cd^2+^]_F_ reached 3.06×10^-10^ M. In addition, both cyanobacterial strains grew more slowly under the nutrient-limited conditions. Their μ was 0.37, 0.20, and 0.16 d^-1^ for WT and 0.37, 0.07, and 0.19 d^-1^ for MT in the control treatment of the +NP,-P, and—N toxicity tests, respectively. Then we investigated whether nutrient condition and the inactivation of MC synthesis play any considerable roles in the sensitivity of *M. aeruginosa* to Cd. For this purpose, the relative change of μ to its counterpart in the respective control treatment of the six toxicity tests was plotted against [Cd^2+^]_F_ (Fig. 2). [Cd^2+^]_F_-based EC50s (Table 1) were comparable to each other regardless of the nutrient condition and the cyanobacterial strain.

**Table 1:** [Cd^2+^]F-based median effect concentration (EC50) in the nutrient-enriched (+NP), phosphorus-limited (-P), and nitrogen-limited (-N) toxicity tests for *Microcystis aeruginosa* PCC 7806 (WT) and its MC-lacking mutant (MT). Data are mean ± standard error (n = 2)

	**+NP**	**-P**	**-N**
WT	*8.96×10^-11^ ± 4.77×10^-12^	*1.14×10^-10^ ± 4.79×10^-12^	*1.15×10^-10^ ± 3.10×10^-12^
MT	*1.11×10^-10^ ± 1.14×10^-11^	*1.22×10^-10^ ± 5.26×10^-11^	*1.15×10^-10^ ± 7.01×10^-13^

* No significant difference (*p* > 0.05) between these EC50s.

According to FIAM, the toxicity of a metal is determined by its free ion concentration in the experimental medium [5]. Despite the wide acceptance of this model, many exceptions were observed and other types of metal concentration (e.g., cell-surface-adsorbed or intracellular metal concentration) were found to have better toxicity predictability than free metal ion concentration. Miao and Wang [24, 26] reported that different nutrient-conditioned marine phytoplankton are inhibited to different extent at similar free metal ion concentration. This phenomenon cannot be explained by FIAM and is attributable to discrepant metal accumulation or subcellular distribution under these nutrient conditions. Namely, metal toxicity is better predicted by its intracellular concentration and by its concentration in the cytosol. As for freshwater phytoplankton, their intracellular metal to phosphorus ratio (e.g., Cd/P) serves as a better biomarker of its toxicity regarding the effects of phosphorus on metal detoxification and bioaccumulation [11, 29]. In the present study, although the—P cyanobacteria accumulated less Cd than its +NP and—N counterparts (Fig. 1a), they had similar [Cd^2+^]_F_-based EC50s (Table 1). Our results seem to be in contrast to the findings of Wang and Dei [29] as well as Zeng and Wang [11]. Nevertheless, Cd toxicity to WT and MT was still dependent on Cd/P, as will be further clarified below. The good predictability of [Cd^2+^]_F_ was just because of the synchronous decrease in [Cd]_intra_ together with the cellular concentration of TP.

### Cd detoxification in *M. aeruginosa*

Phytoplankton can adopt several strategies to alleviate metal toxicity. First, they can reduce metal accumulation by uptake regulation or efflux acceleration [30]. For instance, the transporters of the green alga *S. obliquus* have higher affinity, but lower capacity to arsenate than those of *C. reinhardtii* [31]. Consequently, the former alga can tolerate arsenate better than the latter. Besides the manipulation of metal transporters in the plasma membrane, phytoplankton can also excrete a variety of organic matter (e.g., polysaccharides and protein-like substances) to the environment [32, 33]. These substances reduce the ambient concentration of free metal ion and lower its internalization according to FIAM. Secondly, the subcellular distribution of metal can be altered to minimize its interactions with sensitive intracellular sites. For instance, Lavoie et al. [34] found that the fraction of Cd in organelles decreases while its proportion in granules increases with the accretion of [Cd^2+^]_F_. Further, phytoplankton can synthesize various biomolecules like Poly-P [35, 36], carbohydrates [23, 37, 38], and LMW thiols [34, 39, 40] inside the cells. With the help of these molecules, the noxious metals are scavenged into relatively inert subcellular compartments (e.g., vacuoles and metal-rich granules). In addition, MC, as a secondary metabolite of cyanobacteria, was also proposed to be associated with metal detoxification via the preceding pathways (e.g., metal complexation) [2, 7].

### TP and Poly-P

**Figure 3:**
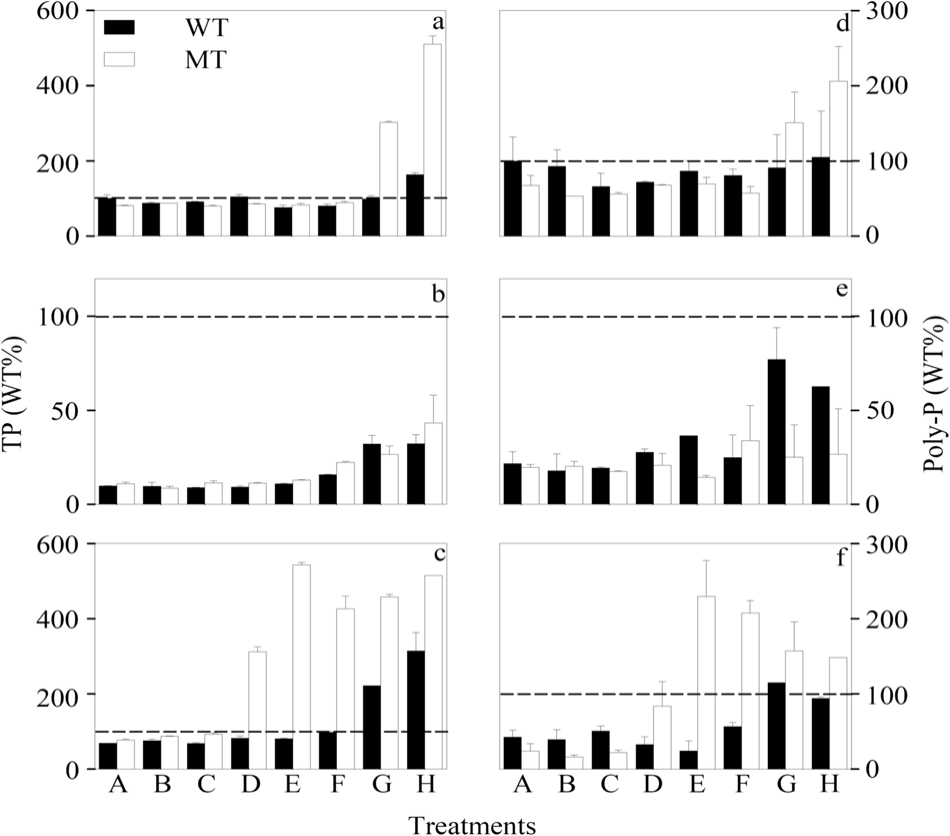
Cellular concentration of (a-c) total phosphorus (TP) and (d-f) inorganic polyphosphate (Poly-P) in treatments A-H of the (a, d) nutrient-enriched (+NP), (b, e) phosphorus-limited (-P), and (c, f) nitrogen-limited (-N) toxicity tests for *Microcystis aeruginosa* PCC 7806 (WT, black bar) and its MC-lacking mutant (MT, white bar). All values were normalized to levels (100% as represented by the dashed lines) detected in the WT strain at the lowest respective Cd concentration (Treatment A). Cd concentration in treatments A-H ([Cd]_T_, 1.00×10^-8^—9.95×10^-6^ M; [Cd^2+^]_F_, 1.00×10^-13^—1.21×10^-8^ M) is listed in Table B of S1 File. Data are mean ± standard error (n = 2)

At the end of each toxicity test, cellular TP concentration was first kept constant, but increased significantly (*p* < 0.05, two-way ANOVA, Table C in S1 File) at higher [Cd^2+^]_F_, even when its value was lowered by one order of magnitude under the—P condition (Fig. 3 and Figure A in S1 File). Consequently, the cellular TP concentration of WT was enhanced by 63.6%, 230.5%, and 358.1% in treatment H, compared to the control treatment of the +NP,-P, and—N toxicity tests, respectively. The stimulative effect of Cd on cellular TP indicates that phosphorus might play an important role in alleviating Cd toxicity to WT and MT. This hypothesis was further supported by our findings that the +NP,-P, and—N cells displayed comparable Cd/P at the same [Cd^2+^]_F_ regardless of the nutrient conditions and cyanobacterial strain (Fig. 1b). As [Cd^2+^]_F_-based EC50s of the six toxicity tests were similar to each other (Table 1), Cd toxicity was still determined by intracellular Cd/P in the present study. Therefore, the good predictability of [Cd^2+^]_F_ was just attributable to the synchronous decrease of [Cd]_intra_ together with the cellular concentration of TP. Phosphorus association with Cd detoxification may partly explain why WT and MT with comparable cellular concentration of TP at lower levels of Cd had similar sensitivity to Cd. However, the underlying mechanisms why MT showed higher cellular concentration of TP than WT at higher levels of [Cd^2+^]_F_ (Table C in S1 File), especially under the +NP and—N conditions, need to be further investigated.

Inorganic polyphosphate is a chain of tens to hundreds of phosphate residues linked by high-energy phosphoanhydride bonds [41]. Metal chelating is one of its functions inside cells [42]. In the present study, Poly-P had a significant contribution to TP, especially under the—P condition (Figure A in S1 File). Approximately 17.6–36.7%, 26.8–101.9%, and 7.93–21.1% of TP was accounted for by Poly-P in all treatments of the +NP,-P, and—N toxicity tests of MT, respectively. Similarly, Poly-P makes up 80% of TP in the green alga *Chlorella vulgaris* [19]. A substantial amount of Poly-P (0.045 mg P/g dry weight cells) was also found in the cytoplasm of *M. aeruginosa* by electron microscopy [43]. Further, the variation of cellular Poly-P concentration with [Cd^2+^]_F_, nutrient conditions, and cyanobacterial strain displayed the same pattern as that of TP (Fig. 3 and Table C in S1 File). Hence, part of the detoxification effects of cellular phosphorus might come from Poly-P, which can alleviate metal toxicity at least in two ways. On one hand, Poly-P can sequester metals directly inside the cells, as supported by its co-localization with nickel ion in the cyanobacterium *Anacystis nidulans* R2 [44]. On the other hand, Nishikawa et al. [45] found that cellular Poly-P concentration decreases together with a substantial increase in vacuolar orthophosphate content when *C. reinhardtii* is exposed to 20 μM Cd. They further proposed that Poly-P degradation into orthophosphate is more important than its cellular level in metal detoxification. Although Poly-P also exists in marine phytoplankton [46], its association with metal detoxification of these organisms has not been observed. This may be one of the reasons why phosphorus has negligible effects on metal accumulation by marine phytoplankton in contrast to their freshwater counterpart [24, 26].

### Intra- and extracellular carbohydrates

**Figure 4:**
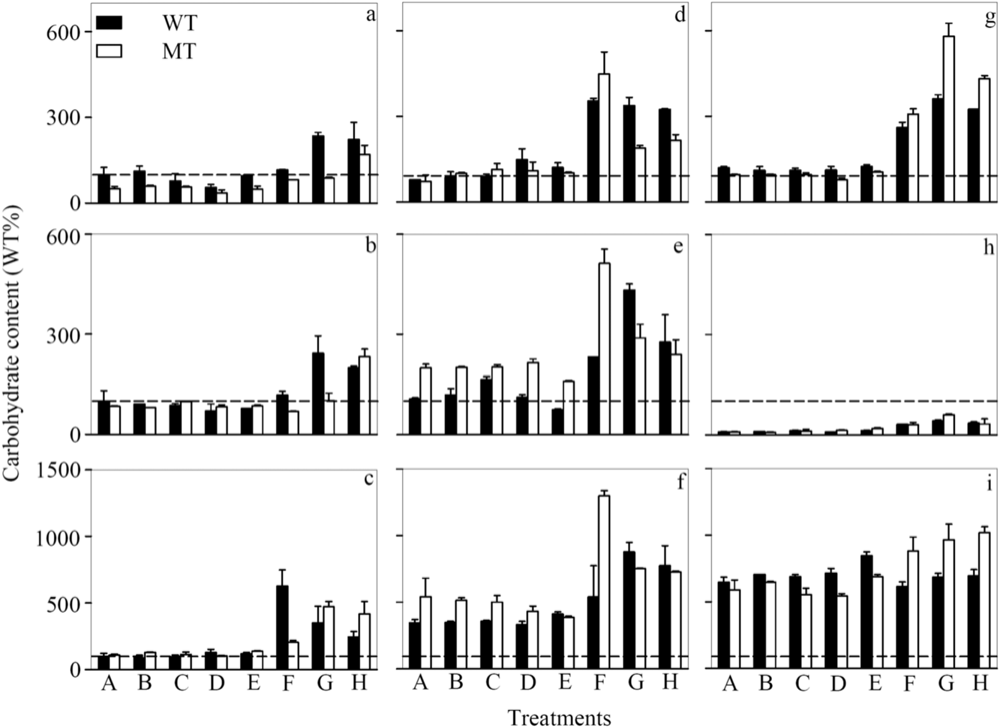
Cell-volume-normalized concentration of (a, d, g) monosaccharide and (b, e, h) polysaccharide excreted by the cells as well as (c, f, i) cellular concentration of carbohydrates retained inside the cells in the (a-c) nutrient-enriched (+NP), (d-f) phosphorus-limited (-P), and (g-i) nitrogen-limited (-N) toxicity tests for *Microcystis aeruginosa* PCC 7806 (WT, black bar) and its MC-lacking mutant (MT, white bar). All values were normalized to levels (100% as represented by the dashed lines) detected in the WT strain at the lowest respective Cd concentration (Treatment A). Cd concentration in treatments A-H ([Cd]_T_, 1.00×10^-8^—9.95×10^-6^ M; [Cd^2+^]_F_, 1.00×10^-13^—1.21×10^-8^ M) is listed in Table B of S1 File. Data are mean ± standard error (n = 2)

Being the major component of exopolymeric substances, carbohydrates play an important role in metal biogeochemical cycling and detoxification [47]. In the present study, Cd not only induced the synthesis or slowed the degradation of carbohydrates in *M. aeruginosa* cells but also accelerated their excretion into the experimental medium (Fig. 4 and Figure B in S1 File). In the +NP toxicity test for WT, there was 1.23, 0.99, and 1.45 time significant (*p* < 0.05, one-way ANOVA) enhancement in [CHO]_mono_, [CHO]_poly_, and [CHO]_cell_, respectively, when [Cd^2+^]_F_ went up from 1×10^-13^ M in the control treatment to 1×10^-9^ M in treatment H. Up-regulated production of extracellular polysaccharides was previously found to be a general response to Cu, Cd, and Ag in various marine phytoplankton [23, 37]. Moreover, the polysaccharides of the cyanobacterium *Anabaena spiroides* have a high binding affinity to Mn, Cu, Pb, and Hg [48]. In particular, alginic acid as a typical polysaccharide is able to reduce metal accumulation by the American oyster (*Crassostrea virginica*) [49]. These findings suggest that carbohydrates could mitigate metal toxicity through chelation. Nevertheless, other possibilities like the relief of metal-induced oxidative stresses by carbohydrates cannot be excluded [50]. Based on the results of two-way ANOVA (Table C in S1 File), [CHO]_mono_, [CHO]_poly_, and [CHO]_cell_ displayed significant (*p* < 0.05) difference between WT and MT. However, no consistent trend was found either for the different nutrient-conditioned *M. aeruginosa* or for the same nutrient-conditioned cells at different [Cd^2+^]_F_. This phenomenon implies strong interactions between MC, nutrient limitation, and ambient Cd concentration, which need to be further investigated.

Besides Cd and MC, phytoplankton nutrient status also influenced carbohydrate metabolism. Total carbohydrate concentration, as the sum of [CHO]_mono_, [CHO]_poly_, and [CHO]_cell_, didn’t change significantly (*p* > 0.05, two-way ANOVA) with nutrient condition. However, the proportion of [CHO]_cell_ to total carbohydrate concentration increased in the following the order: the +NP cells (15.8–51.0%) < the-P cells (28.7–50.7%) < the—N cells (66.0–90.1%) (Figure C in S1 File). Accordingly, the proportion of [CHO]_poly_ significantly (*p* < 0.05, two-way ANOVA, Table C in S1 File) reduced from 45.6–77.8% under the +NP condition to 43.9–68.4% in the—P cells and further to 5.07–19.8% when the cyanobacterium was nitrogen-limited (Figure A in S1 File). In contrast to [CHO]_cell_ and [CHO]_poly_, the proportion of [CHO]_mono_ stayed low (< 15%) in all treatments with no considerable effects from phytoplankton nutrient condition. Elevated [CHO]_cell_ in nutrient-limited cyanobacteria has been reported before [51, 52]. When the marine diatom *Chaetoceros affinis* approaches its stationary phase with nitrate depleted in the medium, its cellular carbohydrate content increases by one order of magnitude [51]. Likewise, Lynn et al. [52] also found that [CHO]_cell_ is higher in the Si-, P- or N-limited freshwater diatom *Stephanodiscus minutulus*. These phenomena imply that carbon fixation is less influenced by nutrient starvation and thus more carbohydrates are formed when the carbon fixed cannot be utilized in the synthesis of other biomolecules (e.g., protein and lipid). Nevertheless, the possibility that fewer carbohydrates are degraded under the nutrient-limited condition cannot be excluded. Although there were more carbohydrates in the nutrient-limited cells, fewer carbohydrates were liberated. This was in contrast to the previous finding that nitrogen and phosphorus limitation favors the release of carbohydrates [53]. Therefore, nutrient effects on carbohydrate liberation might be phytoplankton species specific. The reduced [CHO]_poly_ possibly resulted from the down-regulation of the enzymes (e.g., glucanase) involved in the transformation of cellular glucan to extracellular carbohydrates [54].

### Cellular LMW thiols

**Figure 5:**
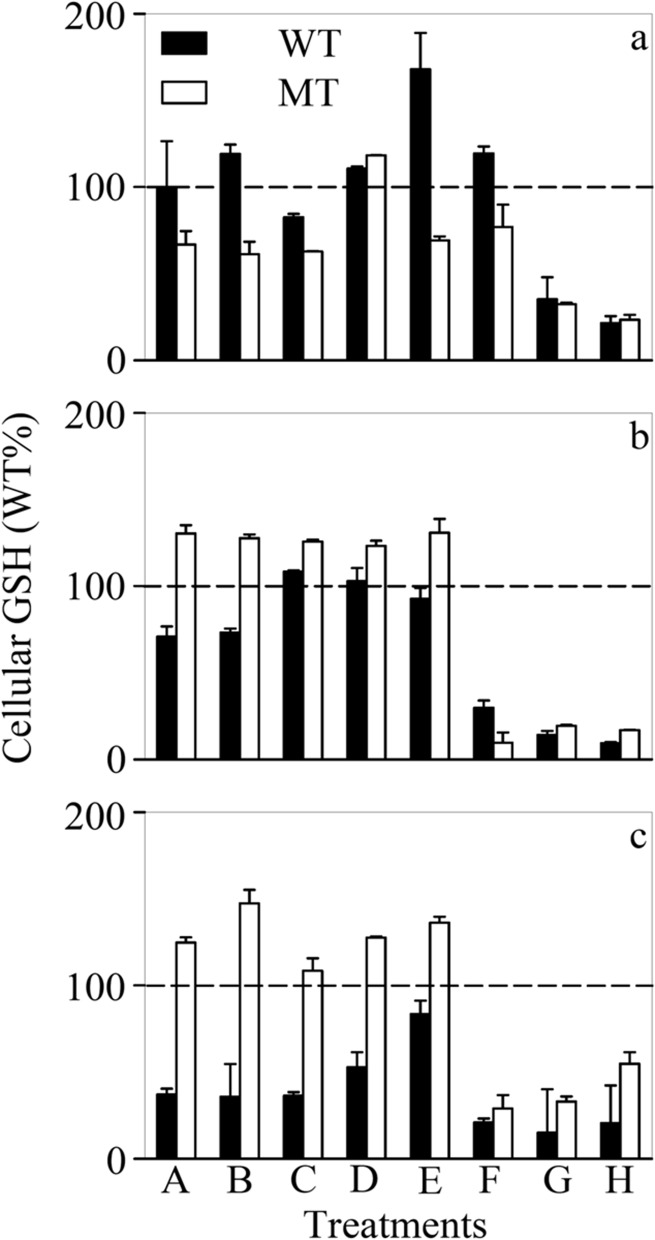
Cellular concentration of glutathione ([GSH]_cell_) in treatments A-H of the (a) nutrient-enriched (+NP), (b) phosphorus-limited (-P), and (c) nitrogen-limited (-N) toxicity tests for *Microcystis aeruginosa* PCC 7806 (WT, black bar) and its MC-lacking mutant (MT, white bar). All values were normalized to levels (100% as represented by the dashed lines) detected in the WT strain at the lowest respective Cd concentration (Treatment A). Cd concentration in treatments A-H ([Cd]_T_, 1.00×10^-8^—9.95×10^-6^ M; [Cd^2+^]_F_, 1.00×10^-13^—1.21×10^-8^ M) is listed in Table B of S1 File. Data are mean ± standard error (n = 2)

LMW thiols such as GSH and phytochelatins [(γ-Glu-Cys)_n_-Gly, n = 2–4] have been demonstrated to be involved in metal complexation and detoxification in eukaryotic phytoplankton [55]. As for WT and MT, their [GSH]_cell_ decreased strikingly (e.g., as much as 86.6–89.4% under the—P condition) at higher levels of Cd (Fig. 5 and Figure D in S1 File). Such abrupt reduction might be explained by two possibilities. First, GSH could remove reactive oxygen species and thus more GSH was consumed at higher [Cd^2+^]_F_ to alleviate the oxidative stresses caused by Cd [56]. Second, GSH might be transformed to other LMW thiols (e.g., phytochelatins), which could scavenge trace metals in the vacuoles of phytoplankton [57]. Although WT contained significantly (*p* < 0.05, two-way ANOVA, Table C in S1 File) more GSH than MT under the +NP condition, a reverse trend was observed when the cells were N or P limited, suggesting different extent of oxidative stresses for these two cyanobacterial strains under nutrient limitation. On the other hand, phytochelatins were below the detection limit in present study suggesting that *M. aeruginosa* was unable to synthesize these thiols in appreciable amounts. Our finding further supports the previous opinion that cyanobacteria lack the phytochelatin synthase gene observed in eukaryotes [58]. Although suspicious genes with similar functions were reported in cyanobacteria, one of the proteins (Alr0975), encoded in the genes of the cyanobacterium *Nostoc* sp. does not possess the phytochelatin synthase ability [59].

Despite the absence of phytochelatins in *M. aeruginosa*, other LMW thiols [e.g., (γ-Glu-Cys)_n_-βAla, (γ-Glu-Cys)_n_-Ser and (γ-Glu-Cys)_n_-Glu] might be formed in this cyanobacterium accompanying the depletion of GSH [60, 61]. On the other hand, [GSH]_cell_ of nutrient-limited WT was significantly (*p* < 0.05, two-way ANOVA) lower than their +NP counterpart while a reverse trend was observed for MT. Such discrepancy between WT and MT may be attributable to the oxidative stresses triggered by nutrient limitation. Considering the fact that microcystin could possibly alleviate these oxidative stresses, more GSH was thus formed in microcystin-lacking MT [62].

### Microcystin in the cells

**Figure 6:**
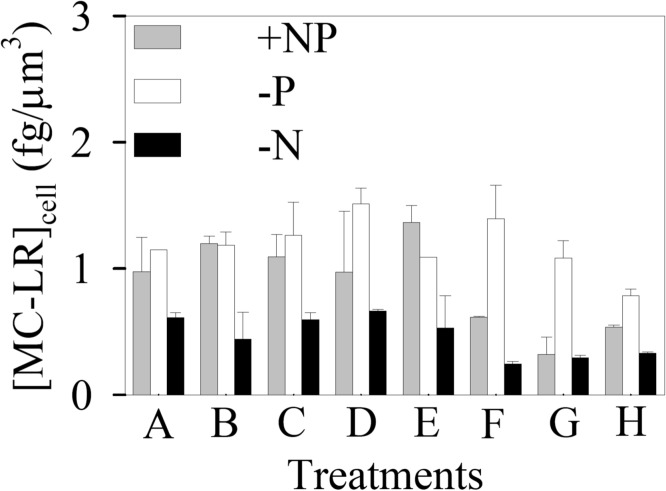
Cellular MC-LR concentration ([MC-LR]_cell_, fg/μm^3^) in treatments A-H of the nutrient-enriched (+NP), phosphorus-limited (-P), and nitrogen-limited (-N) toxicity tests for *Microcystis aeruginosa* PCC 7806 (WT). Cd concentration in treatments A-H ([Cd]_T_, 1.00×10^-8^—9.95×10^-6^ M; [Cd^2+^]_F_, 1.00×10^-13^—1.21×10^-8^ M) is listed in Table B of S1 File. Data are mean ± standard error (n = 2)

Although the adverse effects of MC have drawn considerable attention from the public, its potential function in the cyanobacteria themselves remains largely unknown. In the present study, no MC-LR was detected in MT and [MC-LR]_cell_ of WT stayed constant when [Cd^2+^]_F_ was low, but went down by as much as 44.1% at higher [Cd^2+^]_F_ (Fig. 6). As nitrogen was required during the synthesis of MC, [MC-LR]_cell_ of the—N cells was significantly (*p* < 0.05) lowered. Nevertheless, the—N cyanobacterium displayed similar sensitivity to Cd (EC50 = 1.15×10^-10^ M), compared to its +NP counterpart (EC50 = 8.96×10^-11^ M) (Table 1). Although MT doesn’t possess any variant of MC, its EC50 was similar to that of WT under each nutrient condition and both strains also had comparable [Cd]_intra_. All these phenomena suggest that MC might not have any direct effects on the intracellular complexation or detoxification of Cd. The hypothesis was supported by the study of Fujii et al. [8], where the uptake of both ferric and ferrous iron by *M. aeruginosa* PCC 7806 remains unchanged when the MC synthesis gene *mcyH* in this cyanobacterium is inactivated. Our previous study also manifests that 1 mg/L MC does not have any considerable roles in the bioavailability of various metals (Cd, Cu, Zn, and Cr) to the green alga *C. reinhardtii* [4].

In summary, despite the lower Cd accumulation under the—P condition, the [Cd^2+^]_F_-based of EC50s of the +NP,-P, and-N cells were comparable to each other. This inconsistency was ascribed to the synchronous decrease of [Cd]_intra_ together with the cellular concentration of TP. Hence, Cd/P still had a good predictability of Cd toxicity in the present study. Moreover, our results did show that inactivating MC peptide synthetase gene of *M. aeruginosa* had some nutrient and Cd concentration dependent effects on TP, Poly-P, carbohydrates, and LMW thiols. Nevertheless, both cyanobacterial strains had comparable [Cd]_intra_ and [Cd^2+^]_F_-based EC50s with similar sensitivity to Cd. Based on these findings, we conclude that MC cannot bind strongly with metals and has no effects on metal toxicity. Other possible functions of MC need to be further investigated.

## Supporting Information

S1 File This file contains supporting Tables A, B, C, and Figures A, B, C, D. **Table A.** Chemical components and their concentrations in BG-11_m_.
**Table B.** Total dissolved ([Cd]_T_, mol/L) and free Cd ion ([Cd^2+^]_F_, mol/L) concentrations in treatments A-H of the six toxicity tests including two cyanobacterial strains (*Microcystis aeruginosa* PCC 7806 and its microcystin-lacking mutant) and three nutrient conditions [nutrient-enriched (+NP), phosphorus-limited (-P), and nitrogen-limited (-N) conditions].
**Table C.** Effects of microcystin (MC), Cd concentration, and nutrient limitation on the cellular concentration of total phosphorus (TP), inorganic polyphosphate (Poly-P), glutathione (GSH), and microcystin (MC) as well as on the concentration of cellular carbohydrates ([-CHO]_cell_) and the concentration of mono- ([-CHO]_mono_) and polysaccharide ([-CHO]_poly_) excreted by the cyanobacteria in the nutrient-enriched (+NP), phosphorus-limited (-P), and nitrogen-limited (-N) toxicity tests based on the results of two-way (MC vs. Cd concentration or Nutrient status vs. Cd concentration) ANOVA.
**Figure A.** Actual value of cellular concentration of (a-c) total phosphorus (TP) and (d-f) inorganic polyphosphate (Poly-P) in treatments A-H of the (a, d) nutrient-enriched (+NP), (b, e) phosphorus-limited (-P), and (c, f) nitrogen-limited (-N) toxicity tests for *Microcystis aeruginosa* PCC 7806 (WT, black bar) and its MC-lacking mutant (MT, white bar). Cd concentration in treatments A-H ([Cd]_T_, 1.00×10^-8^—9.95×10^-6^ M; [Cd^2+^]_F_, 1.00×10^-13^—1.21×10^-8^ M) is listed in Table B of S1 File. Data are mean ± standard error (n = 2).
**Figure B.** Actual value of cell-volume-normalized concentration of (a, d, g) monosaccharide and (b, e, h) polysaccharide excreted by the cells as well as (c, f, i) cellular concentration of carbohydrates retained inside the cells in the (a-c) nutrient-enriched (+NP), (d-f) phosphorus-limited (-P), and (g-i) nitrogen-limited (-N) toxicity tests for *Microcystis aeruginosa* PCC 7806 (WT, black bar) and its MC-lacking mutant (MT, white bar). Cd concentration in treatments A-H ([Cd]_T_, 1.00×10^-8^—9.95×10^-6^ M; [Cd^2+^]_F_, 1.00×10^-13^—1.21×10^-8^ M) is listed in Table B of S1 File. Data are mean ± standard error (n = 2).
**Figure C.** The proportion of [CHO]_mono_ (black bar), [CHO]_poly_ (white bar), and [CHO]_cell_ (gray bar) to the concentration of total carbohydrate produced by *Microcystis aeruginosa* PCC 7806 (WT, first column) and its microcystin-lacking mutant (MT, second column) in the (a) nutrient-enriched (+NP), (b) phosphorus-limited (-P), and (c) nitrogen-limited (–N) toxicity tests, respectively. Data are mean ± standard error (n = 2).
**Figure D.** Actual value of cellular concentration of glutathione ([GSH]_cell_) in treatments A-H of the (a) nutrient-enriched (+NP), (b) phosphorus-limited (-P), and (c) nitrogen-limited (-N) toxicity tests for *Microcystis aeruginosa* PCC 7806 (WT, black bar) and its MC-lacking mutant (MT, white bar). Cd concentration in treatments A-H ([Cd]_T_, 1.00×10^-8^—9.95×10^-6^ M; [Cd^2+^]_F_, 1.00×10^-13^—1.21×10^-8^ M) is listed in Table B of S1 File. Data are mean ± standard error (n = 2).
(DOC)
