# Supplementary material for: Cadmium Toxicity to Microcystis aeruginosa PCC 7806 and Its Microcystin-Lacking Mutant
Source: PLoS One. 2015 Jan 15;10(1):e0116659. doi: 10.1371/journal.pone.0116659 (PMC4295860; doi:10.1371/journal.pone.0116659)
Supplement: S1 File — Table A. Chemical components and their concentrations in BG-11m. Table B. Total dissolved ([Cd]T, mol/L) and free Cd ion ([Cd2+]F, mol/L) concentrations in treatments A-H of the six toxicity tests including two cyanobacterial strains (Microcystis aeruginosa PCC 7806 and its microcystin-lacking mutant) and three nutrient conditions [nutrient-enriched (+NP), phosphorus-limited (-P), and nitrogen-limited (-N) conditions]. Table C. Effects of microcystin (MC), Cd concentration, and nutrient limitation on the cellular concentration of total phosphorus (TP), inorganic polyphosphate (Poly-P), glutathione (GSH), and microcystin (MC) as well as on the concentration of cellular carbohydrates ([-CHO]cell) and the concentration of mono- ([-CHO]mono) and polysaccharide ([-CHO]poly) excreted by the cyanobacteria in the nutrient-enriched (+NP), phosphorus-limited (-P), and nitrogen-limited (-N) toxicity tests based on the results of two-way (MC vs. Cd concentration or Nutrient status vs. Cd concentration) ANOVA. Figure A. Actual value of cellular concentration of (a-c) total phosphorus (TP) and (d-f) inorganic polyphosphate (Poly-P) in treatments A-H of the (a, d) nutrient-enriched (+NP), (b, e) phosphorus-limited (-P), and (c, f) nitrogen-limited (-N) toxicity tests for Microcystis aeruginosa PCC 7806 (WT, black bar) and its MC-lacking mutant (MT, white bar). Cd concentration in treatments A-H ([Cd]T, 1.00×10^-8^—9.95×10^-6^ M; [Cd2+]F, 1.00×10^-13^—1.21×10^-8^ M) is listed in Table B of S1 File. Data are mean ± standard error (n = 2). Figure B. Actual value of cell-volume-normalized concentration of (a, d, g) monosaccharide and (b, e, h) polysaccharide excreted by the cells as well as (c, f, i) cellular concentration of carbohydrates retained inside the cells in the (a-c) nutrient-enriched (+NP), (d-f) phosphorus-limited (-P), and (g-i) nitrogen-limited (-N) toxicity tests for Microcystis aeruginosa PCC 7806 (WT, black bar) and its MC-lacking mutant (MT, white bar). Cd concentration in [file pone.0116659.s001.doc]

**Supporting Information S1 File**

**Cadmium toxicity to *Microcystis aeruginosa* PCC 7806 and its microcystin-lacking mutant**

Bin Huang, Shen Xu, Ai-Jun Miao*, Lin Xiao*, Liu-Yan Yang

State Key Laboratory of Pollution Control and Resource Reuse, School of the Environment, Nanjing University, Nanjing, Jiangsu Province, China

8 pages, 3 Tables, and 4 Figures

*Corresponding authors: A. J. Miao (miaoaj@nju.edu.cn); L. Xiao (xiaolin@nju.edu.cn)

**Table A.** Chemical components and their concentrations in BG-11_m_.

| Components | Final concentration (mg/L) | Components | Final concentration (mg/L) |
| --- | --- | --- | --- |
| citric acid | 6.0 | Na_2_EDTA | 1.0 |
| ferric ammonium citrate | 6.0 | H_3_BO_3_ | 2.86 |
| NaNO_3_ | 1500 | MnCl_2_·4H_2_O | 1.86 |
| K_2_HPO_4_ | 40.0 | ZnSO_4_·7H_2_O | 0.22 |
| MgSO_4_·7H_2_O | 75.0 | CuSO_4_·5H_2_O | 0.079 |
| CaCl_2_·2H_2_O | 27.0 | Na_2_MoO_4_·2H_2_O | 0.39 |
| Na_2_CO_3_ | 36.0 | Co(NO_3_)_2_·6H_2_O | 0.049 |

**Table B.** Total dissolved ([Cd]_T_, mol/L) and free Cd ion ([Cd^2+^]_F_, mol/L) concentrations in treatments A-H of the six toxicity tests including two cyanobacterial strains (*Microcystis aeruginosa* PCC 7806 and its microcystin-lacking mutant) and three nutrient conditions [nutrient-enriched (+NP), phosphorus-limited (-P), and nitrogen-limited (-N) conditions].

| Treatments | +NP | | -P | | -N | |
| --- | --- | --- | --- | --- | --- | --- |
|  | [Cd]_T_ | [Cd^2+^]_F_ | [Cd]_T_ | [Cd^2+^]_F_ | [Cd]_T_ | [Cd^2+^]_F_ |
| A | 1.00×10^-8^ | 1.00×10^-13^ | 1.00×10^-8^ | 1.00×10^-13^ | 1.00×10^-8^ | 1.00×10^-13^ |
| B | 1.00×10^-7^ | 1.11×10^-12^ | 3.00×10^-7^ | 1.13×10^-11^ | 1.20×10^-6^ | 1.06×10^-11^ |
| C | 1.00×10^-6^ | 1.22×10^-11^ | 8.00×10^-7^ | 3.19×10^-11^ | 2.80×10^-6^ | 3.02×10^-11^ |
| D | 2.00×10^-6^ | 2.80×10^-11^ | 2.20×10^-6^ | 1.04×10^-10^ | 4.50×10^-6^ | 6.38×10^-11^ |
| E | 3.10×10^-6^ | 5.04×10^-11^ | 4.50×10^-6^ | 3.05×10^-10^ | 6.00×10^-6^ | 1.11×10^-10^ |
| F | 5.00×10^-6^ | 1.11×10^-10^ | 7.40×10^-6^ | 1.09×10^-9^ | 8.00×10^-6^ | 3.15×10^-10^ |
| G | 7.30×10^-6^ | 3.06×10^-10^ | 8.90×10^-6^ | 3.06×10^-9^ | 9.35×10^-6^ | 1.13×10^-9^ |
| H | 9.00×10^-6^ | 1.00×10^-9^ | 9.65×10^-6^ | 1.01×10^-8^ | 9.95×10^-6^ | 1.21×10^-8^ |

**Table C.** Effects of microcystin (MC), Cd concentration, and nutrient limitation on the cellular concentration of total phosphorus (TP), inorganic polyphosphate (Poly-P), glutathione (GSH), and microcystin (MC) as well as on the concentration of cellular carbohydrates ([-CHO]_cell_) and the concentration of mono- ([-CHO]_mono_) and polysaccharide ([-CHO]_poly_) excreted by the cyanobacteria in the nutrient-enriched (+NP), phosphorus-limited (-P), and nitrogen-limited (-N) toxicity tests based on the results of two-way (MC vs. Cd concentration or Nutrient status vs. Cd concentration) ANOVA.

| Parameters | MC effects | | | Cd concentration effects | | | Nutrient effects | |
| --- | --- | --- | --- | --- | --- | --- | --- | --- |
|  | +NP | -P | -N | +NP | -P | -N | WT | MT |
| TP | WT < MT | WT = MT | WT < MT | *p* < 0.05 | *p* < 0.05 | *p* < 0.05 | +NP = -N > -P | -N > +NP > -P |
| Poly-P | WT = MT | WT > MT | WT < MT | *p* < 0.05 | *p* = 0.06 | *p* < 0.05 | +NP > -N = -P | -N = +NP > -P |
| [-CHO]_mono_ | WT > MT | WT > MT | WT < MT | *p* < 0.05 | *p* < 0.05 | *p* < 0.05 | -N > -P = +NP | -N > -P > +NP |
| [-CHO]_poly_ | WT > MT | WT < MT | WT = MT | *p* < 0.05 | *p* < 0.05 | *p* < 0.05 | +NP = -P > -N | -P > +NP > -N |
| [-CHO]_cell_ | WT = MT | WT < MT | WT = MT | *p* < 0.05 | *p* < 0.05 | *p* < 0.05 | -N > -P > +NP | -N > -P > +NP |
| GSH | WT > MT | WT < MT | WT < MT | *p* < 0.05 | *p* < 0.05 | *p* < 0.05 | -P = +NP > -N | -P > -N > +NP |
| MC | ---^a^ | --- | --- | *p* < 0.05 | *p* < 0.05 | *p* < 0.05 | -P > +NP > -N | --- |

^a^ No data available.

**Figure A.** Actual value of cellular concentration of (a-c) total phosphorus (TP) and (d-f) inorganic polyphosphate (Poly-P) in treatments A-H of the (a, d) nutrient-enriched (+NP), (b, e) phosphorus-limited (-P), and (c, f) nitrogen-limited (-N) toxicity tests for *Microcystis aeruginosa* PCC 7806 (WT, black bar) and its MC-lacking mutant (MT, white bar). Cd concentration in treatments A-H ([Cd]_T_, 1.00×10^-8^ – 9.95×10^-6^ M; [Cd^2+^]_F_, 1.00×10^-13^ – 1.21×10^-8^ M) is listed in Table B of S1 File. Data are mean ± standard error (n = 2).

**Figure B.** Actual value of cell-volume-normalized concentration of (a, d, g) monosaccharide and (b, e, h) polysaccharide excreted by the cells as well as (c, f, i) cellular concentration of carbohydrates retained inside the cells in the (a-c) nutrient-enriched (+NP), (d-f) phosphorus-limited (-P), and (g-i) nitrogen-limited (-N) toxicity tests for *Microcystis aeruginosa* PCC 7806 (WT, black bar) and its MC-lacking mutant (MT, white bar). Cd concentration in treatments A-H ([Cd]_T_, 1.00×10^-8^ – 9.95×10^-6^ M; [Cd^2+^]_F_, 1.00×10^-13^ – 1.21×10^-8^ M) is listed in Table B of S1 File. Data are mean ± standard error (n = 2).

**Figure C.** The proportion of [CHO]_mono_ (black bar), [CHO]_poly_ (white bar), and [CHO]_cell_ (gray bar) to the concentration of total carbohydrate produced by *Microcystis aeruginosa* PCC 7806 (WT, first column) and its microcystin-lacking mutant (MT, second column) in the (a) nutrient-enriched (+NP), (b) phosphorus-limited (-P), and (c) nitrogen-limited (–N) toxicity tests, respectively. Data are mean ± standard error (n = 2).

**Figure D.** Actual value of cellular concentration of glutathione ([GSH]_cell_) in treatments A-H of the (a) nutrient-enriched (+NP), (b) phosphorus-limited (-P), and (c) nitrogen-limited (-N) toxicity tests for *Microcystis aeruginosa* PCC 7806 (WT, black bar) and its MC-lacking mutant (MT, white bar). Cd concentration in treatments A-H ([Cd]_T_, 1.00×10^-8^ – 9.95×10^-6^ M; [Cd^2+^]_F_, 1.00×10^-13^ – 1.21×10^-8^ M) is listed in Table B of S1 File. Data are mean ± standard error (n = 2).
